# Research progress in computer-aided diagnosis systems for lung cancer

**DOI:** 10.1038/s41746-025-02101-3

**Published:** 2025-11-26

**Authors:** Ke Ma, Min Zheng, Wenli Chen, Yunxiang Qi, Hao Rong

**Affiliations:** 1 Department of Thoracic Surgery, Sichuan Clinical Research Center for cancer, Sichuan Cancer Hospital & Institute, Sichuan Cancer Center, University of Electronic Science and Technology of China, Chengdu, China; 2 Department of Medical Oncology, Sichuan Clinical Research Center for cancer, Sichuan Cancer Hospital & Institute, Sichuan Cancer Center, University of Electronic Science and Technology of China, Chengdu, China; 3 Department of Radiation Oncology, Sichuan Clinical Research Center for cancer, Sichuan Cancer Hospital & Institute, Sichuan Cancer Center, University of Electronic Science and Technology of China, Chengdu, China

**Keywords:** Cancer, Oncology

## Abstract

Lung cancer remains the top cause of cancer death, demanding consistent decisions. This clinically oriented review synthesizes computer-aided diagnosis across classical imaging, machine learning, and deep learning, emphasizing bedside-proven advances: multimodal CT/PET–clinical fusion; small-data strategies; interpretable AI; and privacy-preserving multi-center learning. Reported systems reach AUC ≥ 0.95 with <0.1 false positives/CT and boost early detection by ~20–30%; prognostic C-index ~0.85–0.90. We outline implementation checkpoints and priorities to convert accuracy into patient benefit.

## Introduction

Due to the characteristics of lung cancer, such as atypical early symptoms and rapid biological progression, clinical epidemiological reports show that more than 68% of patients are already in locally advanced Stage III or have metastatic Stage IV when they seek medical attention due to symptoms^1,2^. At this point, the tumor has often invaded the pleura, mediastinum, or has distant metastases, and thus lost the opportunity for surgical treatment. In the past, relying on doctors’ subjective analysis of medical images with the naked eye had the following irreparable limitations: First, sensitivity depends on doctors’ experience^3,4^. The missed detection rate of pulmonary nodules can be as high as 30% for junior, young, or less experienced radiologists. Even for experienced senior radiologists, the missed diagnosis rate for micro-nodules with a diameter of <5 mm can reach 30%^5,6^. Second, pathological biopsy is an essential but highly invasive method for clinical diagnosis of benign and malignant lesions. Biopsy has a false negative rate of 20–30% and can cause complications such as bleeding and pneumothorax^7,8^. Third, multi-center clinical studies have shown that the consistency of diagnoses made by experts from different medical institutions using the same imaging data is only 65–72%, and the direct impact of different diagnoses on clinical treatment and prognosis is it can be imagined^8,9^. The current diagnostic process fully reveals the urgency of introducing intelligent auxiliary tools in lung cancer diagnosis and treatment to further improve the standardization of diagnosis.

From the perspective of clinical practice, an ideal lung cancer detection method should have three basic functions: quickly detecting early lung cancer, that is, having a highly sensitive ability to detect early lung cancer to find small nodule lesions that are easily missed in daily image reading^10^; making correct pathological qualitative judgments on lesions through non-invasive means to reduce invasive operations; and objectively providing risk factors such as disease progression and treatment response during follow-up after lung cancer. Lung cancer CAD systems have gradually evolved from these three aspects, and their ideal goal is to convert unstructured content in medical images into expressible and describable standardized quantitative information, replacing human factors with quantitative and objective indicators^11,12^.

The development of lung cancer CAD systems can be clearly divided into three technical stages, which are the three stages of deep integration with clinical application requirements. From the 1990s to 2010, it was the traditional algorithm stage, where the system used threshold segmentation and manually designed features to complete pulmonary nodule detection^13,14^. Typical algorithms were region-growing-based segmentation methods and template-matching-based detection strategies. Due to insufficient feature expression ability, the sensitivity of the system at this stage was about 70–80%, and the false positive rate was high (>1.0/CT), so clinical application was limited^15^.

The period from 2010 to 2018 was the era of machine learning. The gradual improvement of algorithms such as support vector machines and random forests enabled CAD systems to start applying high-dimensional features to improve diagnosis. At the same time, a typical breakthrough in this period was that feature selection and ensemble learning strategies further improved the diagnostic performance of the system to 0.85–0.90, and the false positive rate of pulmonary nodule detection was further reduced to below 0.5/CT^16,17^, reaching the initial level of clinical assistance. Some systems began to carry out pilot applications in some large medical institutions in European and American countries.

The period from 2018 to 2020 was the deep learning period. The introduction of models such as convolutional neural networks (CNN) and Transformer completed end-to-end diagnosis and changed the technical system of traditional CAD systems. Deep models automatically learn discriminative image features without manual design, making the AUC of the system exceed 0.95^18,19^, and the performance of some tasks is close to or exceeds that of experienced radiologists. At present, it has shifted from single image analysis to the fusion of multi-modal data, and from a single diagnostic function to prognosis prediction and treatment response evaluation, forming a technical system covering the entire lung cancer diagnosis and treatment cycle^20,21^.

## Core technical modules of lung cancer CAD systems

### Evolution of image acquisition and preprocessing technologies

#### Comparison of imaging modality features

**Fig. 1:**
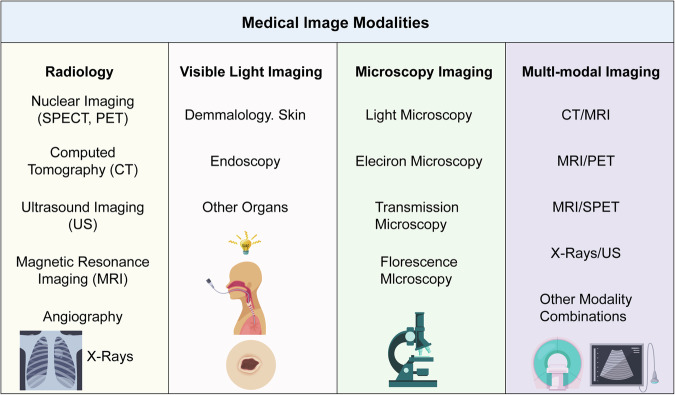
Overview of major medical imaging modalities and representative applications.

Common medical imaging modalities for clinical lung cancer diagnosis each have their own advantages. CAD systems need to select matching modalities or realize multi-modal fusion according to different application scenarios. The comparison chart of different medical imaging modality features is shown in Fig. 1. Computed tomography (CT) has the advantages of high spatial resolution and clear display of lung parenchyma and nodules, and is the mainstream modality of CAD systems^22,23^. The radiation dose of conventional chest CT is usually 5–8 mSv, while LDCT reduces the radiation dose to 1–2 mSv by reducing tube current and tube voltage, and is widely used for lung cancer screening in high-risk groups. The image noise of LDCT increases by about 30%, and special noise removal algorithms are needed for preprocessing, otherwise, it will affect the accuracy of subsequent feature extraction^24,25^.

PET-CT can reflect the metabolic activity of tumors according to the uptake of tracers, so it has high sensitivity (92%) in detecting metastases and is very suitable for lung cancer staging. The standardized uptake value (SUV) of PET-CT can evaluate the malignancy of lesions. Generally, SUVmax > 2.5 suggests malignancy. PET-CT has low spatial resolution and poor ability to display micro-nodules <5 mm. Usually, PET-CT can be combined with CT to complement each other’s advantages^26,27^.

MRI has strong soft tissue resolution, especially for the evaluation of hilar and mediastinal lymph node invasion and chest wall involvement, with an accuracy of 88%, and no radiation damage, which is suitable for people sensitive to radiation. However, MRI has natural difficulties in lung imaging, such as obvious respiratory motion artifacts and long scanning time, so its application in lung cancer CAD is not as extensive as CT, and it is only used as a supplementary modality^28,29^.

Chest X-ray is still used for preliminary screening in primary medical institutions because of its low cost and high popularity, but its detection rate for nodules <10 mm is only 55–65%, and it cannot provide three-dimensional structural information. Its application in CAD systems is gradually marginalized^30,31^, and it is more used as an initial screening tool for joint screening.

This figure summarizes four major categories of medical imaging. Radiology includes nuclear imaging (SPECT, PET), CT, ultrasound, MRI, angiography, and X-rays, which remain the cornerstone of structural and functional diagnosis at the organ and system levels. Visible light imaging, such as dermatology and endoscopy, enables direct real-time visualization of tissues and early disease detection. Microscopy imaging, including light, electron, transmission, and fluorescence microscopy, provides cellular and molecular insights critical for pathology and mechanistic studies. Multi-modal imaging, such as CT/MRI, MRI/PET, MRI/SPET, and X-rays/US, integrates complementary information to improve diagnostic accuracy, lesion characterization, and precision therapy. Together, these modalities highlight the continuum from macroscopic anatomy to microscopic resolution, supporting early detection, individualized treatment, and comprehensive evaluation in clinical and research practice.

#### Key preprocessing technologies

The preprocessing steps of contemporary lung cancer CAD systems have been optimized many times, including reducing image noise, standardizing image quality, emphasizing image features, etc., to facilitate subsequent analysis. The first module is denoising. For the noise characteristics of LDCT, the non-local means (NL-means) filtering technology can significantly reduce noise without losing edge information by means of weighted averaging of matched similar image blocks^32,33^, and the peak signal-to-noise ratio increases by 10–15 dB. In recent years, deep learning-based denoising methods such as DnCNN and BM3D have achieved higher advantages than traditional methods^34,35^. Under the same noise level, the structural similarity index (SSIM) increases by 0.05–0.08.

Lung parenchyma segmentation is a key link in preprocessing, which segments the lung part of chest CT images to avoid the influence of interference factors. A hybrid scheme of U-Net deep learning segmentation and morphological operations is mainly adopted. First, U-Net is used to obtain the initial segmentation mask, and then morphological operations are used to remove non-lung parenchyma areas to obtain the segmentation result^36,37^. The Dice coefficient can reach 0.98 ± 0.01, which can basically meet the expected clinical requirements. However, for complex cases with severe emphysema and atelectasis, an attention mechanism is introduced to focus on the lung parenchyma boundary, which improves the segmentation accuracy by 5–8%.

Respiratory motion correction. Respiratory artifacts caused by respiratory movement in chest CT scanning can be registered between images of different respiratory phases by using 4D-CT registration. Among them, lung parenchyma segmentation is first performed for each phase of 4D-CT, and then the deformation registration algorithm is used to align each phase to the reference phase. Therefore, the volume measurement error caused by motion artifacts does not exceed 3%^38^, which is very important for judging the measurement of nodule growth rate and formulating radiotherapy plans.

Gray normalization can eliminate the gray deviation caused by different devices and different scanning parameters. Usually, Z-score standardization is used to map the image gray to a distribution with a mean of 0 and a standard deviation of 1, so that the gray distribution range of different cases is consistent, and the coefficient of variation is below 5%. For multi-center data, histogram matching technology will be additionally introduced to force the gray distribution of images from different centers to be as similar as possible, so as to improve the cross-center generalization of the model.

The latest research on image enhancement technology based on generative adversarial network (GAN) shows that GAN can improve the diagnostic value of low-dose images. Cycle GAN can make LDCT images comparable to conventional CT images^39,40^, thus increasing the sensitivity of subsequent nodule detection by 8–10% without increasing the false positive rate, which is conducive to promoting LDCT screening.

In clinical workflows, parameter choices for window conditioning and intensity normalization should be predefined and stress-tested for robustness. For routine lung nodule detection/radiomics, clipping to a lung-parenchyma window (e.g., $-\mathrm{1000,400}$HU) reduces outlier leverage and improves cross-scanner comparability; an auxiliary mediastinal window (e.g., $-\mathrm{150,250}$HU) can be added when assessing chest-wall/mediastinal involvement. Intensity normalization is recommended within a lung mask using Z-score scaling (mean 0, SD 1). For radiomics, the discretization scheme must be fixed across sites (e.g., fixed bin width of 25 HU), because bin width materially alters gray-level co-occurrence and run-length features. In multi-center settings, optional histogram matching or feature-level harmonization (e.g., ComBat) may mitigate protocol/device shifts, but any such step should be version-locked and fully documented to ensure reproducibility.

Registration and resampling parameters directly affect downstream feature stability. Longitudinal CT–CT alignment should begin with rigid/affine registration and escalate to deformable B-spline or diffeomorphic methods when respiration or posture varies, with a target registration error <1–2 mm verified by landmarks or overlap metrics on vessels/airways; for PET–CT fusion, pair SUV normalization with deformable alignment for accurate metabolic–anatomic co-localization. Resampling to isotropic voxels stabilizes 3D morphology/texture: 1.0–1.5 mm suits most nodules, while 0.75–1.0 mm is advisable for sub-centimeter lesions or radiotherapy planning; use B-spline/linear interpolation for images and nearest-neighbor for masks. To quantify robustness, we implement a parameter-sensitivity protocol that perturbs each setting within clinically plausible ranges (e.g., ±20% voxel size; ±10–20 HU bin width; rigid vs. deformable registration) and report test–retest or ICC, targeting ICC ≥ 0.85 for key radiomic/deep features before model lock. A brief checklist should report: window/clipping ranges, normalization mask/method, discretization rule and bin width, registration class and acceptance criteria, resampling spacing and interpolators, and any cross-site harmonization applied.

#### Comparative overview of segmentation and radiomics toolchains

To support reproducible lung cancer imaging analytics, we summarize commonly used open-source tools that appeared earlier in the manuscript—ITK-SNAP, 3D Slicer, and PyRadiomics—and outline their strengths, limitations, and best-fit scenarios across screening, diagnosis, and treatment-planning workflows (Table 1).

**Table 1:** Comparison of commonly used medical image analysis tools: primary functions, strengths, limitations, and representative applications

Tool	Primary function	Key strengths	Limitations	Representative scenarios	References
ITK-SNAP	Interactive 3D segmentation and annotation (semi-automatic active contour + manual tools)	Fast, precise voxel-wise delineation with intuitive UI; excellent for lesion boundary refinement; exports NIfTI/MetaImage; good intra-/inter-rater QA	ITK-SNAP	Interactive 3D segmentation and annotation (semi-automatic active contour + manual tools)	^88^
3D Slicer	Extensible imaging platform (Segmentation, Registration, DICOM, RT toolkits)	Rich modules (Segment Editor, SlicerRT, registration); strong DICOM support; scripting via Python; plugins (e.g., MONAI Label) enable AI-assisted auto-segmentation; good for multi-step pipelines [Fedorov et al., 2012] (europepmc.org)	3D Slicer	Extensible imaging platform (Segmentation, Registration, DICOM, RT toolkits)	^89^
PyRadiomics	Radiomics feature extraction (IBSI-aligned)	Standardized feature definitions; YAML-configurable; batch processing; harmonization-friendly; integrates with Python ML stacks [Fornacon-Wood et al., 2020] (SpringerLink)	PyRadiomics	Radiomics feature extraction (IBSI-aligned)	^90^
Segmentation variability & radiomics robustness (note: cross-tool segmentation)	–	Some studies show shape features robust across segmentation, others show high sensitivity for texture / first-order features to segmentation variation [Belfiore et al., 2022] (MDPI)	Segmentation variability & radiomics robustness (note: cross-tool segmentation)	–	^91^

**Applicable scenarios and recommendations**.

1. Ground-truth segmentation & small-cohort studies: Start in ITK-SNAP for precise manual/semi-auto masks; export masks and meta-data for QA logs.

2. End-to-end research pipelines/RT planning: Use 3D Slicer to chain segmentation, registration, and (if needed) dose context (SlicerRT); pin Slicer version and save scenes to ensure reproducibility.

3. Radiomics at scale/multi-center generalization: Standardize resampling/binning and extract IBSI-aligned features with PyRadiomics (YAML configuration under version control). Apply feature harmonization (e.g., ComBat) when scanners/protocols vary; report resampling, bin width, and mask provenance alongside AUC/C-index, calibration, and DCA.

4. Custom preprocessing or site standardization: Implement intensity normalization, LDCT-specific denoising, or deformable registration with SimpleITK/ITK and call from Slicer or standalone Python.

In addition to conventional software tools such as PyRadiomics and 3D Slicer, multimodal image fusion is essential for clinical applications where complementary strengths of different imaging modalities must be combined. For PET/CT, CT provides high-resolution anatomical information while PET captures metabolic activity; accurate integration requires SUV normalization, resampling PET to CT voxel grids, and rigid or deformable registration with a target registration error within 1–2 mm. 3D Slicer with SlicerElastix offers an end-to-end pipeline for DICOM import, registration, and quality assurance, while PyRadiomics ensures standardized IBSI-compliant feature extraction on aligned PET and CT datasets. When large-scale or more sophisticated registration is required, scripted packages such as ANTs or Elastix can provide flexible control. These combined workflows ensure robust PET/CT integration for radiomics or AI-based diagnostic systems.

For MRI + CT fusion, challenges primarily arise from MRI’s intensity inhomogeneity and susceptibility distortions compared with CT. Preprocessing steps such as N4 bias-field correction and modality-specific intensity normalization are recommended prior to multimodal alignment. Mutual-information–based rigid or affine registration, followed by deformable refinement if necessary, is generally performed with 3D Slicer or ANTs/Elastix, with accuracy assessed by Dice similarity and landmark-based TRE (target <2 mm). PyRadiomics can then be applied to extract modality-specific features using harmonized resampling and discretization settings. Overall, 3D Slicer provides strong advantages for clinical usability and visualization, while ANTs/Elastix offer superior flexibility in challenging MRI + CT registration tasks. Together, these tools enable reliable integration of anatomical and functional imaging, thereby improving the reproducibility and translational value of multimodal radiomics studies.

### Feature engineering and representation learning

#### Manually designed feature system

Manually designed features are the features of traditional CAD systems, which complete the function of identifying lung cancer by quantitatively describing the morphological, texture, density features, etc., of lesions. These features describe the characteristics of lesions from multiple angles, and the established feature system is relatively perfect. In terms of morphological features, sphericity is an index to identify malignant nodules. Literature points out that the sphericity of most malignant nodules is <0.7, while that of benign nodules is mostly > 0.9^41^; the lobulation index is calculated according to the concavity and convexity of nodule edges, and >0.3 indicates the possibility of malignancy; the number of spicules reflects the invasiveness of tumors, and the malignant probability of nodules with ≥3 spicules is 4.2 times that of nodules without spicules.

**Fig. 2:**
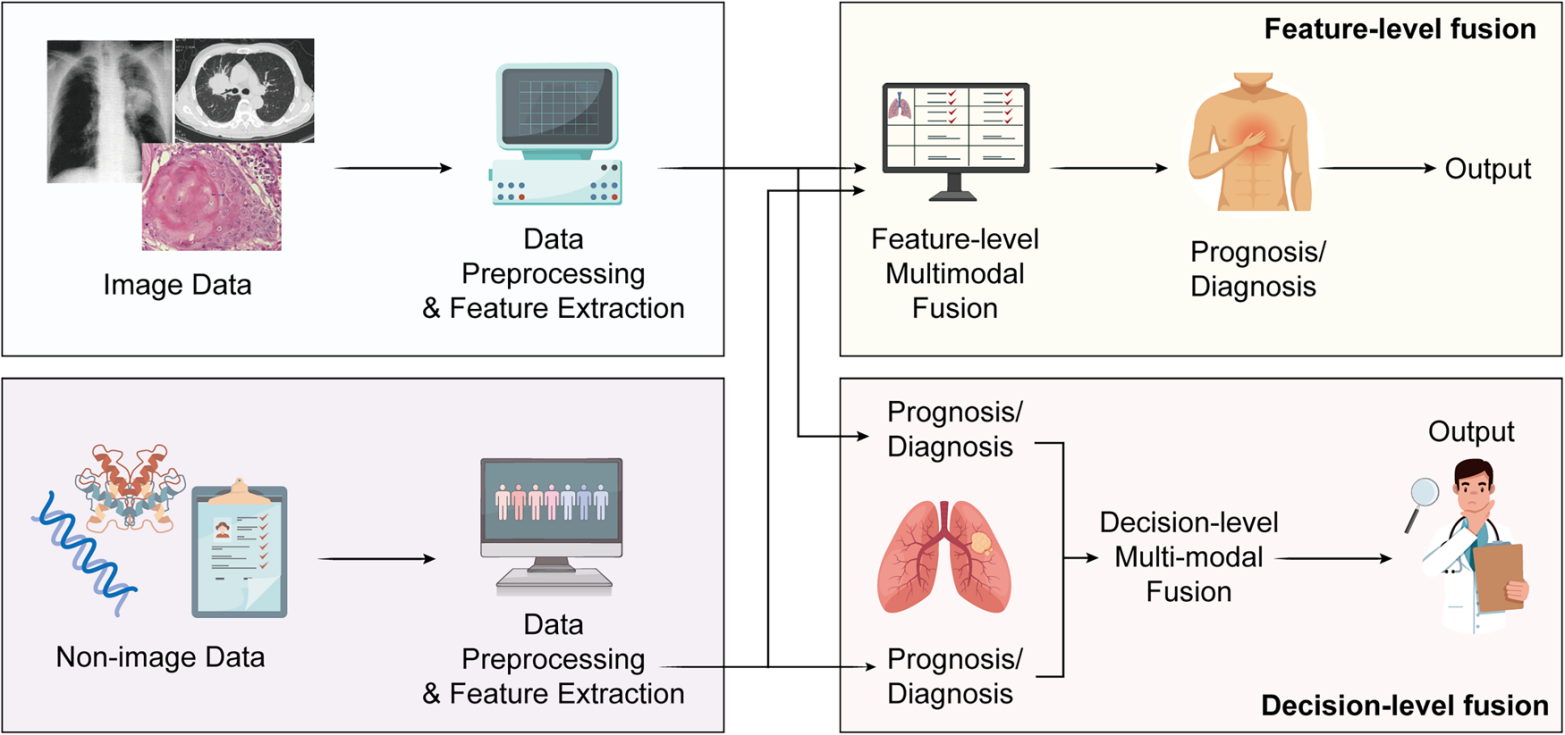
Framework of multimodal data fusion strategies in medical prognosis and diagnosis.

Texture features can measure the spatial distribution of image gray, reveal the microstructural changes of lesions. Contrast and entropy calculated by gray level co-occurrence matrix (GLCM) are commonly used features. Clinical results show that the contrast of malignant nodules is as shown in Fig. 2, and the average contrast is 45 ± 12^42,43^, which is significantly higher than that of benign nodules (28 ± 8); entropy can describe the complexity of gray distribution. The entropy of malignant nodules is greater than that of benign nodules, suggesting that the internal of the lesion is more uneven, and the local binary pattern (LBP) is very sensitive to the change of micro-texture, which helps to distinguish the benign and malignant of ground-glass nodules (GGN).

In CT images, the density characteristics of GGN have special significance. The CT value of pGGN is mostly −600 to −300HU, and it is positively correlated with the degree of invasion, that is, the CT value of pure ground-glass nodules is mostly < −500HU, which are mostly precancerous lesions or minimally invasive adenocarcinomas^44,45^; the higher the proportion of solid components in mixed ground-glass nodules and the higher the CT value, the deeper the invasion degree, and the postoperative recurrence rate increases accordingly.

Growth rate is a dynamic feature, mainly manifested as the growth characteristics of nodules, volume doubling time (VDT), which is calculated according to CT images at different time points. Studies have confirmed that the VDT of malignant nodules is mostly <400 days, while that of benign nodules is mostly >600 days^46,47^. The VDT of lung adenocarcinoma is about 100–400 days, while that of small cell lung cancer is shorter. The combined application of these features, using logistic regression or SVM classification, can improve the accuracy of benign and malignant identification to 82–88%.

This figure illustrates the workflow of integrating image and non-image data for clinical decision support. Image data, such as radiology and pathology, undergo preprocessing and feature extraction before entering multimodal fusion pipelines. Similarly, non-image data—including molecular, genomic, and clinical records—are processed to extract key features. Two major strategies are applied for integration. In feature-level fusion, features from multiple modalities are combined at an early stage, allowing the model to capture complementary structural, molecular, and clinical patterns for joint prediction of prognosis and diagnosis. In decision-level fusion, modality-specific models produce individual predictions, which are then aggregated to form a final output. This approach enhances robustness by leveraging strengths of each modality while reducing bias from single data sources.

#### Deep learning feature learning

**Fig. 3:**
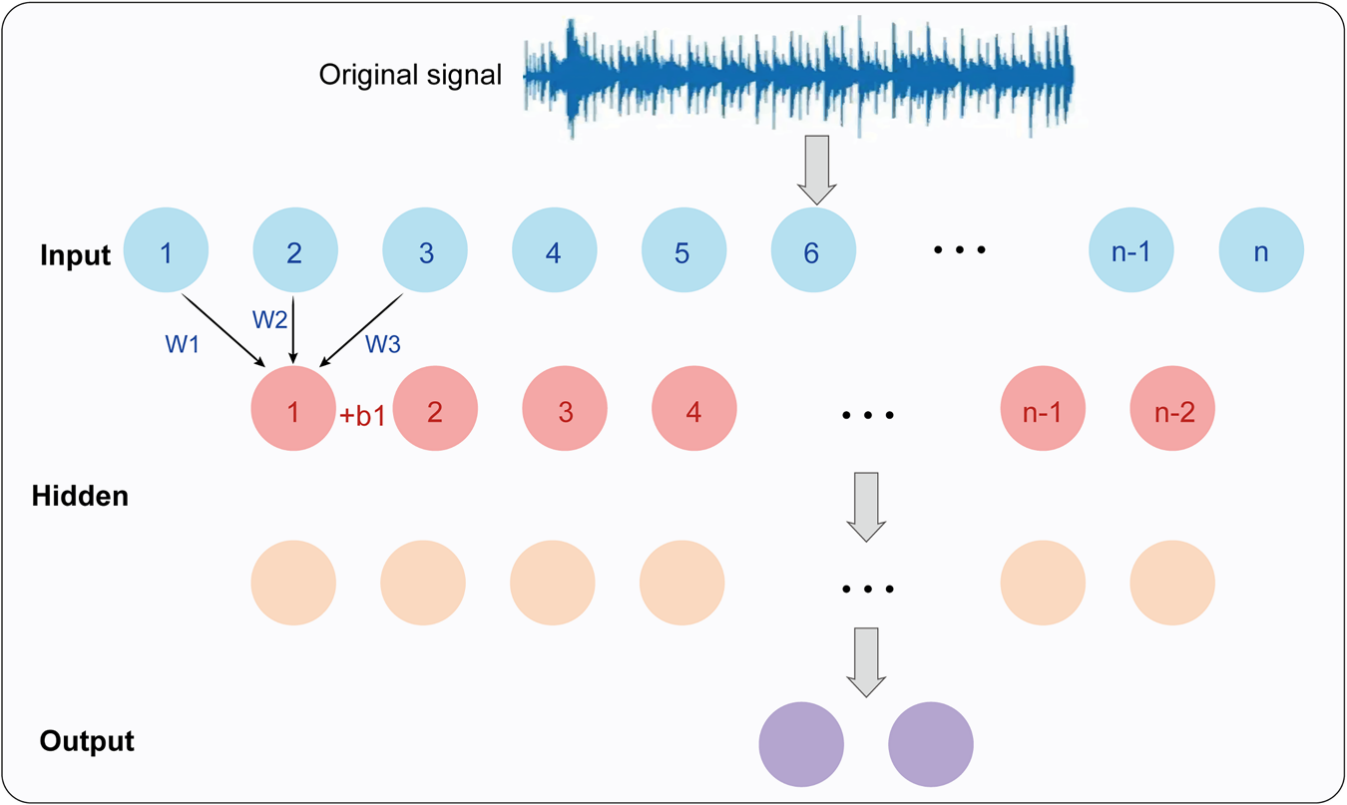
Schematic representation of signal processing through a neural network architecture.

Deep learning has subverted the traditional feature extraction mode. Based on multi-layer nonlinear transformation, it automatically learns the discriminative features of images without manual design, and its ability to express features is stronger than that of manual features. The typical architecture of feature learning is convolutional neural network (CNN), which uses a hierarchical feature extraction mechanism, as shown in Fig. 3, which is very similar to the human visual system: the convolution kernel of the shallow layer (1–3 layers) extracts low-level features such as edges and textures, nodule boundary clarity, and internal density changes; layers 4–6 combine low-level features to find the morphological features of nodules, such as lobulation and spiculation^48,49^; the deep layer extracts more abstract semantic features and establishes the relationship between the pathological attributes of lesions.

In order to strengthen the pertinence of feature learning, an attention mechanism is also introduced. The convolutional block attention module (CBAM) uses channel attention and spatial attention to dynamically adjust the weights on different feature maps, so that the model automatically pays attention to important diagnostic areas such as nodule edges, pleural traction, and vascular convergence, and the feature weight is 2–3 times larger than that of other areas^50^. In the lung nodule classification task, the AUC value of the ResNet50 model with CBAM is 0.04–0.06 higher than that of the basic model.

Contrastive learning trains on positive and negative sample pairs. Images of different phases of the same patient are used as positive samples, and images of different patients are used as negative samples. The feature representation learned by the trained model is more robust^51^. Through contrastive learning training, the feature distance between nodules of the same type is reduced by 40%, and the feature distance between nodules of different types is increased by 35%, which is very effective in the case of small samples.

A large number of studies have shown that the diagnostic performance based on deep features is significantly better than that based on manual features. The deep features extracted by ResNet50 for the identification of benign and malignant lung nodules can reach an AUC value of 0.93, which is significantly higher than the AUC of 0.86 for the optimal combination of manual features^52^; especially, it has significant advantages in distinguishing atypical nodules, which can improve the accuracy by 12–15%. This is due to the fact that deep features can extract some local patterns that cannot be perceived by the human eye, such as the distribution law of solid components in GGN and the distance relationship with surrounding blood vessels.

Although convolutional networks demonstrate strong performance in automated feature extraction, their decision-making process often lacks transparency in clinical practice. Current interpretive approaches attempt to highlight the regions most responsible for a prediction, thereby linking deep features with recognizable radiological signs such as spiculation or pleural traction. These overlays can help radiologists verify whether the model is focusing on clinically relevant structures rather than background artifacts. Nevertheless, several shortcomings remain: the highlighted regions may extend into adjacent vessels or bronchi, the saliency can shift with scanning protocols or reconstruction kernels, and subtle density gradients in ground-glass nodules are often underrepresented. Such inconsistencies limit reproducibility and weaken confidence in the clinical validity of deep features.

To address these limitations, recent studies have explored methods that combine multi-scale attention mechanisms, vessel-aware constraints, and weak supervision from radiologist-marked regions to ensure that the emphasized features are consistent with known pathological cues. Other strategies include integrating quantitative metrics of faithfulness and perturbation-based validation, ensuring that the emphasized regions are not only visually plausible but also causally related to the model’s decision. These improvements are essential to transform CNN-based representations from abstract image patterns into clinically interpretable evidence that can be reliably adopted in diagnostic workflows.

This figure illustrates the transformation of an original signal into predictive outputs via layered neural computations. The input layer receives sequential elements derived from the raw signal, each represented as discrete units. Weighted connections (W1, W2, W3) and bias terms (e.g., +b1) modulate the contribution of each input to the subsequent hidden layer, enabling the network to capture temporal dependencies and nonlinear relationships. Hidden layers progressively abstract the input features, mapping them into higher-level representations that preserve critical patterns while filtering noise. The architecture processes multiple time steps in parallel, aligning with recurrent or feed-forward strategies commonly used in sequence modeling. Finally, the output layer integrates these transformed features to generate predictions or classifications, such as signal interpretation, pattern recognition, or diagnostic decisions. Overall, the diagram highlights the core principles of neural network learning—weighted summation, bias adjustment, nonlinear transformation, and hierarchical feature extraction—that underlie modern signal analysis in artificial intelligence applications.

### Decision models and inference mechanisms

#### Traditional machine learning models

Before the widespread application of deep learning, traditional machine learning models were the decision core of CAD systems. Different models are suitable for different scenarios due to their algorithm characteristics. Support vector machine (SVM) realizes binary classification by finding the optimal classification hyperplane. It performs stably in small sample scenarios. When using radial basis function (RBF) kernel function, the classification accuracy of benign and malignant lung nodules reaches 85%, and it is not sensitive to noise data. However, its performance is greatly affected by the quality of features. When the feature dimension exceeds the sample size, it is easy to overfit.

Random forest reduces the overfitting risk of a single decision tree by integrating the prediction results of 300–500 decision trees, and has strong robustness to missing values and outliers. On multi-center data, the diagnostic consistency Kappa coefficient of random forest is 0.08–0.12 higher than that of SVM, which is more suitable for processing clinical data from complex sources^53,54^. Its feature importance ranking function can also provide some interpretability for doctors, such as outputting the contribution of features such as “spiculation sign” and “lobulated shape”.

Gradient boosting tree captures the interaction between features well by iteratively optimizing weak classifiers, and is often used to fuse clinical features and image features, which demonstrates significant improvement in algorithms that integrate clinical and imaging features. For example, XGBoost fuses 10 image features and 5 clinical features, and the prediction accuracy of lung cancer postoperative recurrence can reach 83%, which is 7 percentage points higher than that of the pure image feature model. However, gradient boosting trees are very sensitive to parameter tuning, and it often takes a lot of experiments to find a suitable parameter combination. In addition, their training time on high-dimensional sparse data is long.

However, traditional models are limited by the expression ability of manual features, and the accuracy decreases by 15–20% in complex cases (such as multiple nodules, cases with severe basic lung diseases), which also promotes the development of deep learning decision models.

#### Deep learning decision models

Deep learning decision models complete the end-to-end mapping from images to diagnostic results, unlike traditional models that separate feature engineering and classifiers. The two-stage detection model is the mainstream architecture of lung nodule detection models. The first stage uses a region proposal network (RPN) to propose nodules with a recall rate >95%. The second stage classifies and corrects the bounding box, and the final F1 value is 0.92, with a false positive rate <0.1/CT. Among them, YOLOv8 has an inference speed of 30 frames per second, which fulfills the real-time requirements for clinical deployment.

Data-efficient strategies for end-to-end CNNs. End-to-end models are sensitive to dataset size, site heterogeneity, and label noise; therefore, we recommend a pragmatic, layered strategy. (i) Transfer learning: initialize with weights pretrained on large image corpora or, preferably, self-supervised pretraining on unlabeled chest CT/PET-CT (e.g., contrastive or masked-image objectives) to learn modality-specific priors; fine-tune with discriminative learning rates (lower for early layers) and progressive unfreezing to avoid catastrophic forgetting. (ii) Class imbalance: use class-balanced sampling and asymmetric/focal losses in conjunction with calibrated thresholds; report minority-class recall and precision alongside AUC. (iii) Regularization & calibration: employ stochastic depth/dropout, weight decay, and temperature scaling or deep ensembles to counter overconfident outputs in scarce-data regimes. (iv) Semi-supervised learning: leverage large pools of unlabeled scans via consistency regularization or pseudo-labeling under strong augmentations, with confirmation by a small expert-reviewed subset. All experiments should adopt patient- and site-level splits, document preprocessing/versioning, and include external validation to verify generalization beyond the source cohort.

Augmentation and synthetic imaging (GAN/diffusion) to overcome data scarcity. Beyond standard geometric transforms, CT-specific augmentations should mimic real acquisition variability (e.g., HU jitter within physical bounds, reconstruction-kernel simulation, Poisson noise for low-dose, mild motion blur, domain randomization of slice thickness) and preserve labels for both classification and segmentation heads. MixUp/CutMix (3D) and lesion-aware cropping/rim augmentation improve sample efficiency for small nodules and peri-tumoral habitats. For rare phenotypes, generative models (GAN or diffusion) can synthesize site-conditional images or lesion inserts to rebalance training, and can support style transfer/domain adaptation across scanners; to mitigate bias, we require: (a) generator training on held-out sites separate from evaluation, (b) feature-level audits (e.g., Fréchet distance/embedding drift) to confirm pathology-consistent realism, and (c) ablations showing gains beyond classical augmentations. When sharing data is restricted, federated learning with on-premise training plus feature harmonization provides multi-center variability without exchanging raw images. We suggest a short reporting checklist for end-to-end studies: pretraining source/objective; augmentation catalog and ranges; imbalance strategy; uncertainty/calibration method; synthetic data usage and safeguards; split protocol (patient/site level); and external calibration/decision-curve analyses before clinical deployment.

The segmentation-classification joint model completes tumor segmentation and qualitative diagnosis synchronously. It extracts features by sharing an encoder, outputs a segmentation mask in the decoder branch, and outputs a diagnostic result in the classification branch^55,56^. The advantage of this architecture is that the segmentation result provides spatial constraints for classification, making the model pay more attention to the features of the tumor area. The Dice coefficient and AUC value reach 0.96 and 0.97 respectively, which is especially suitable for the diagnosis of GGN with blurred boundaries.

Despite the rapid advancement of deep learning architectures such as U-Net and its variants, lung tumor segmentation in clinical practice remains challenging due to inherent image ambiguities. First, blurred tumor boundaries are common, especially in ground-glass nodules and infiltrative lesions, where the margin between lesion and normal parenchyma is indistinct. This often leads to under-segmentation or over-segmentation, even when Dice similarity coefficients exceed 0.9 in benchmark datasets. Second, the anatomical proximity of lung tumors to vascular and airway structures introduces the problem of overlapping intensity distributions. Tumors that abut or encase pulmonary vessels are particularly prone to false boundary delineation, as standard convolutional filters may misinterpret vascular structures as part of the lesion. Third, heterogeneity in background lung diseases, such as emphysema or fibrosis, further complicates segmentation by altering tissue density and mimicking tumor margins. Addressing these challenges requires advanced strategies, including attention-enhanced U-Net models, multi-scale feature aggregation, and vessel-aware segmentation modules that explicitly incorporate anatomical priors to separate vascular structures from tumor tissue. Emerging work combining CT with functional PET uptake maps has also shown promise in resolving boundary ambiguity by jointly leveraging morphological and metabolic cues.

The multi-task model realizes multiple related tasks such as detection, classification, and staging in the same network. It improves computational efficiency through parameter sharing, and the inference speed is 40% faster than that of the single-task model. The multi-task model improved based on Mask R-CNN can output the position, benign/malignant nature, and diameter of pulmonary nodules at the same time^57^. Compared with the single-task model, the processing efficiency is increased by 1.7 times under the same computing resources.

Finally, a tentative measure is the application of the federated learning framework to address the challenges of privacy protection in the above-mentioned multi-center data sharing. By training models locally in each center and only sharing model parameters instead of raw data, the performance of the finally aggregated global model is about 10–15% better than that of the single-center model. A multi-center study found that the sensitivity of the pulmonary nodule detection model using federated learning on the external validation set was 96.8%, which was about 4.2% higher than the average of the individual models in each center.

## Innovation and breakthrough of key technical methods

### Cross-modal data fusion technology

Traditional single-modal images cannot fully describe the biological characteristics of lung cancer. Multi-modal information fusion can utilize the complementary advantages between different modalities to improve the ability of differential diagnosis. The flow chart of multi-modal data fusion technology is shown in Fig. 3. Multi-modal data fusion can fuse data at three levels to form a complete fusion technology system. The lowest level of fusion is data-level fusion, which mainly solves the problem of spatial alignment of images from different modalities. The fusion scheme first completes the rigid transformation through the image registration algorithm to correct the differences in translation and rotation, and then processes the non-linear deformation through elastic transformation to obtain a final spatial error of <1 mm^58^, so as to accurately align the PET-CT metabolic information with the CT anatomical structure. It provides a basis for the later research on the “morphology-metabolism” relationship of tumors.

Feature-level fusion between modalities adopts a weighted fusion method, and the weights are dynamically set according to the discriminative ability of the modalities. Taking the identification of benign and malignant pulmonary nodules as an example, setting the weight of CT morphological features at 0.6–0.7 is more conducive to showing nodule edges, density, etc., and setting the weight of PET metabolic features at 0.3–0.4 is more conducive to showing tumor activity. Automatically learning the weight distribution based on the attention mechanism can increase the AUC value of the fused features by 0.05–0.08 compared with single-mode (CT/PET)^59^. Clinically, fusion can also effectively deal with the problems of “same disease with different images” and “different diseases with same images”. For example, for inflammatory nodules with atypical CT manifestations and early lung cancer, combining PET SUV can significantly improve the discrimination accuracy.

Integrated fusion at the decision level: At the higher fusion decision of the decision level, multi-source information is fused through methods such as Bayesian networks or ensemble learning, including image diagnosis results, gene detection results, and clinical indicators. For example, when predicting the response of lung cancer patients to targeted therapy, the combined analysis of the changing trend of tumor size in CT images, the changing trend of metabolic activity in PET^60^, and the EGFR mutation status can achieve a prediction accuracy of 89%, which is much higher than 65–75% predicted by any single indicator. Many studies have shown that the three-modal integrated fusion model of CT + PET + clinical data can achieve a sensitivity of 98.2% and a specificity of 96.5% for early lung cancer diagnosis, which also has also received solid assistance at the clinical decision-making level.

Rigorous validation is indispensable for ensuring that CAD models for lung cancer achieve not only high accuracy in development cohorts but also stable performance in diverse clinical settings. Internal cross-validation provides an estimate of model robustness, while external validation on independent multi-center datasets serves as the gold standard for generalizability. However, reporting only sensitivity and specificity is insufficient; a comprehensive set of performance indices is required to guide clinical translation.

Among these, the area under the receiver operating characteristic curve (AUC) remains the most widely used metric for classification tasks, reflecting global discrimination ability. For prognostic and survival models, the concordance index (C-index) is recommended to quantify agreement between predicted and observed outcomes. Calibration curves are essential to evaluate the consistency between predicted risk probabilities and actual event rates, addressing potential over- or underestimation. In parallel, decision curve analysis (DCA) provides insight into clinical utility by quantifying net benefit across different threshold probabilities. The combination of these metrics enables a multidimensional assessment of diagnostic accuracy, prognostic reliability, and practical decision-making value, thereby establishing a more solid evidence base for clinical adoption of CAD systems.

### Small sample and imbalanced data processing

**Fig. 4:**
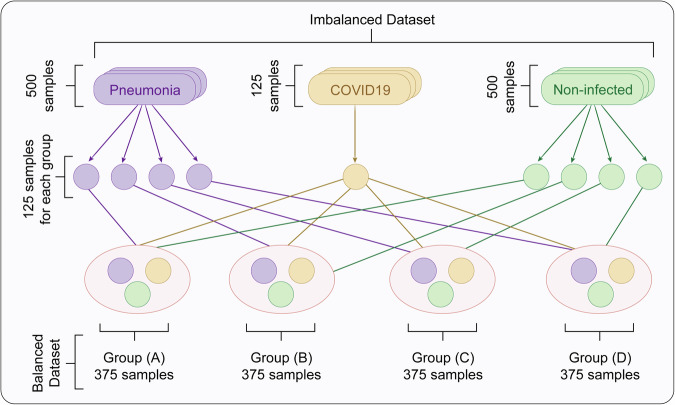
Strategy for constructing a balanced dataset from an imbalanced sample distribution.

The training of lung cancer CAD systems faces severe data challenges. On the one hand, the number of high-quality annotated medical data samples is limited, especially for rare pathological types. On the other hand, the ratio of benign to malignant samples in the dataset is usually 7:3, with a serious class imbalance problem^61^. To address these challenges, researchers have developed a series of solutions, forming a complete technical chain. The technical roadmap for small sample and imbalanced data processing is shown in Fig. 4.

Data augmentation uses realistic and pathologically characteristic synthetic samples to expand the dataset. Traditional methods such as rotation, scaling, and flipping generate basic sample transformations but fail to capture the pathological characteristics of rare cases. The method of GAN is used to generate images of rare cases with pathological features. StyleGAN generates CT images of pulmonary sarcomatoid carcinoma, with morphological and density features that are 92% similar to real samples^62^, increasing the number of training samples by 3–5 times and improving the accuracy of the model in identifying rare subtypes by 12%. GAN variants based on attention can better control the key features of generated samples to improve the quality of augmented data.

The weighted loss function has been validated as an effective solution to class imbalance. FocalLoss assigns smaller weights to easily classified samples and larger weights to difficult-to-classify samples, making the model training pay more attention to small-class samples. Experimental verification shows that the recall rate of malignant samples in the model using FocalLoss increases from 72 to 89%, and the accuracy of benign samples remains above 90%^63^. For extremely imbalanced situations, it can also be combined with oversampling and undersampling to balance the ratio of positive and negative samples before using the weighted loss, which has a better effect.

Transfer learning effectively alleviates the small sample problem. Transferring the model parameters pre-trained on large-scale natural image datasets to medical image tasks enables the model to achieve excellent performance even in small sample situations. The comprehensive scheme of data augmentation + weighted loss + transfer learning provides ideas for dealing with the lack of medical data.

Radiomics for noninvasive profiling of the tumor immune microenvironment (TIME). Contemporary CT- and PET/CT-based radiomics can approximate TIME phenotypes by capturing intra- and peritumoral heterogeneity (texture, edge sharpness, vascular/perivascular patterns) that correlate with immune biomarkers such as PD-L1 expression, CD8⁺ T-cell infiltration, and tumor mutational burden (TMB). Multiple studies report that pre-treatment imaging signatures predict PD-L1 status with clinically useful discrimination and generalizability, and PET/CT deep-learning radiomics further improves PD-L1 prediction compared with clinical baselines. Likewise, volumetric CT radiomics has been validated to estimate TMB and to stratify patients’ likelihood of benefiting from ICIs. Together, these results support the use of standardized, IBSI-aligned feature extraction (with fixed resampling/binning) to derive reproducible, site-agnostic TIME surrogates that can be audited and externally validated^64^.

Imaging biomarkers for ICI benefit prediction and response monitoring. Beyond static biomarkers, baseline and early-on-treatment radiomics signatures—especially those integrating peri-tumoral “rim” features and PET-metabolic habitats—have shown value for forecasting durable clinical benefit/overall response and progression-free survival under anti-PD-1/PD-L1 therapy in NSCLC. Recent works demonstrate that combining radiomics with minimal clinical variables yields higher prognostic performance than clinical models alone; PET/CT-based models are particularly informative when metabolic–anatomic fusion is available. For implementation, we recommend reporting a prespecified timepoint (e.g., pre-ICI baseline), a primary endpoint (e.g., DCB/ORR or PFS), fixed preprocessing parameters, and external validation with calibration and decision-curve analysis; where feasible, multi-center harmonization and model explainability (e.g., SHAP/region-level saliency) should be included to enhance credibility and facilitate clinical adoption^65^.

This figure demonstrates the process of balancing an imbalanced dataset containing pneumonia, COVID-19, and non-infected cases. The original dataset includes 500 pneumonia samples, 500 non-infected samples, and only 125 COVID-19 samples. To ensure equal representation, 125 samples are drawn from each class and redistributed into four balanced groups (A–D), each with 375 samples. This resampling strategy reduces class bias, prevents overrepresentation of certain categories, and improves model robustness. By forming multiple balanced groups, the method supports fair training and validation, ensuring reliable and unbiased diagnostic performance in medical AI applications.

### Model interpretability methods

**Fig. 5:**
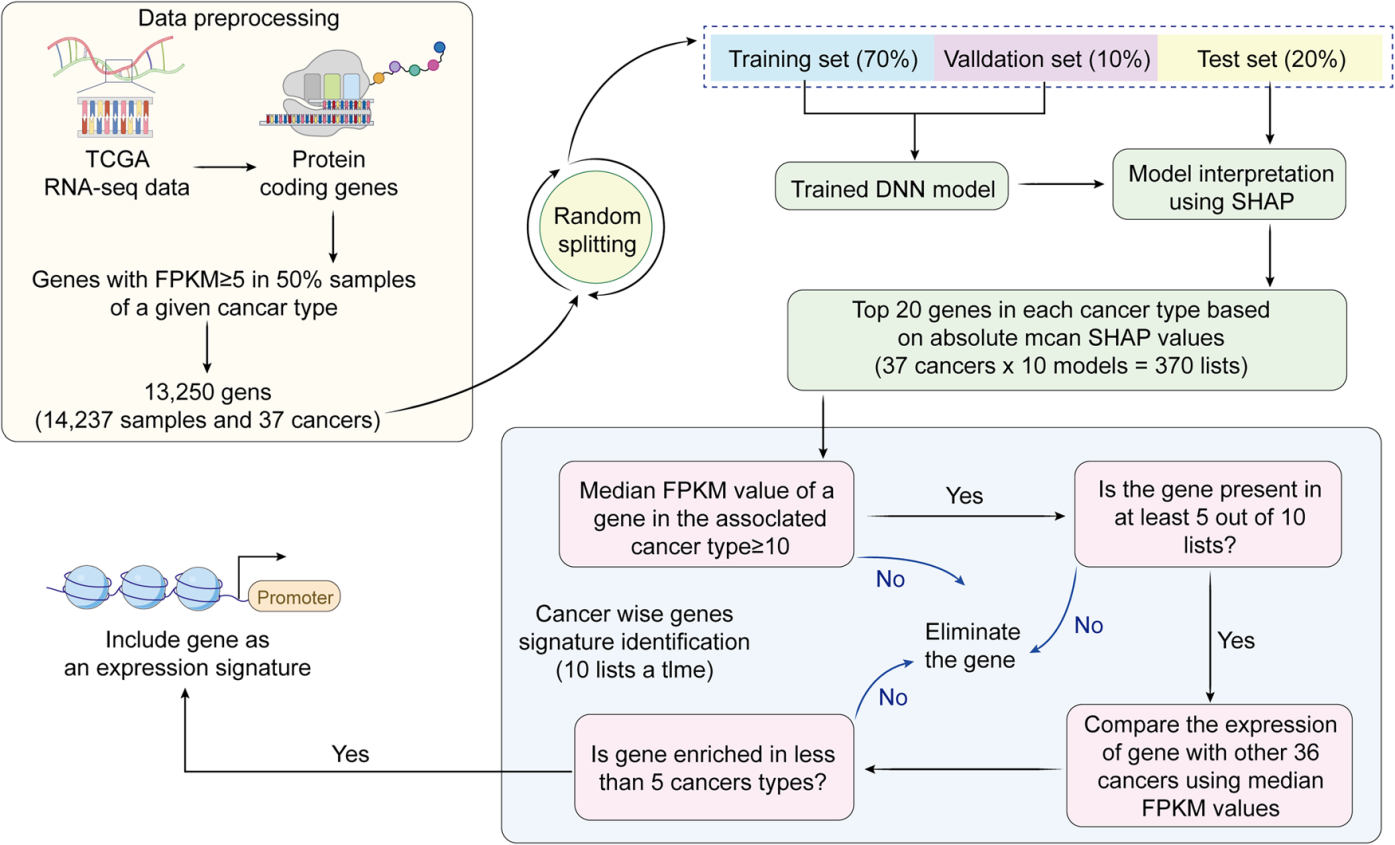
Workflow for gene signature identification using deep learning and SHAP interpretation.

The “black box” nature of deep learning models is one of the main obstacles to their clinical application. Doctors need to understand the basis for the model’s diagnostic decisions to trust and use them reasonably. In recent years, model interpretability technology has made significant progress, forming a multi-level explanation system. The use of deep learning models and SHAP values to explain the model is shown in Fig. 5.

Clinically actionable clustering of imaging subtypes and their molecular correlates. Beyond methodological descriptions, unsupervised clustering of CT (and PET/CT) semantic–radiomic features yields reproducible imaging subtypes—for example, GGO-dominant/pure-GGO, part-solid with a small consolidation core, and solid/invasive phenotypes—that map onto distinct molecular landscapes and clinical behaviors^66^. Meta-analytic and radiogenomic evidence indicates that EGFR-mutant adenocarcinomas are enriched in GGO-dominant and lepidic-pattern clusters, whereas correlations for KRAS are weaker or inconsistent; ALK-rearranged tumors often present as solid masses in younger, light- or never-smokers with characteristic CT appearances (e.g., lobulation), supporting a distinct clustered phenotype. These links have been validated by classical radiology–genomics mapping as well as modern CT radiomics and deep learning–radiomics models that non-invasively predict EGFR mutation status and gene expression programs, with multi-institution studies and recent external validations demonstrating robust performance^67^.

How clustering subtypes guide clinical classification and treatment decisions. First, surgical planning increasingly leverages CT-derived clusters anchored by the consolidation-to-tumor ratio (CTR): part-solid/GGO-dominant clusters (low CTR) identify indolent, lepidic-predominant biology suitable for parenchyma-sparing surgery, whereas solid/invasive clusters (high CTR) signal aggressive behavior requiring wider margins; randomized evidence operationalizes this concept by using thin-section CT criteria to triage segmentectomy vs. lobectomy in cIA disease^68^. Second, targeted-therapy triage benefits from EGFR-enriched clusters: when tissue is limited or biopsy is contraindicated, radiogenomic classifiers support prioritizing plasma genotyping and, where appropriate, early EGFR-TKI initiation; large-scale CT deep learning–radiomics and recent external validation studies reinforce the reliability of EGFR prediction for such decisions^67^. Third, immunotherapy: clustering patients by radiomics-defined tumor habitats and immune-linked features (e.g., peri-tumoral texture/heterogeneity) can stratify likelihood of benefit to ICIs; integrative models that jointly cluster radiologic, clinicopathologic, and transcriptomic signals further improve first-line ICI outcome prediction, offering a pragmatic path to imaging-driven TIME stratification in routine care^69^. For implementation, we recommend reporting the clustering pipeline (feature set, distance metric, stability indices), pre-specifying action thresholds (e.g., CTR cut-points; EGFR-probability for reflex plasma NGS), and providing external calibration/decision-curve analyses before clinical deployment.

The figure shows a workflow for identifying gene expression features. A schematic diagram of the workflow for identifying gene expression features using deep learning models and SHAP values. Feature attribution analysis quantifies the effect of each feature on the diagnostic result. SHAP values calculate the marginal contribution of each feature to the prediction result based on game theory. For example, in a certain case, the SHAP value of “lobulated boundary” is 0.23 ± 0.05^70^, indicating that this feature has a positive contribution to the malignant diagnosis; “smooth boundary” is −0.18 ± 0.04, which is a negative contribution. Such a quantitative explanation allows doctors to clearly understand the main factors of the model’s decision and judge whether it conforms to clinical logic.

The interpretability method performs reverse reasoning on the internal decisions of the model to obtain explanations in natural language. The trained interpretable model converts the model’s feature vector into structured text, such as “The nodule is 92% malignant because there are spiculation signs (0.35), pleural traction (0.28), and SUVmax = 8.5 (0.22)”^71^. Such explanations are consistent with the format of doctors’ diagnosis reports and are easier for doctors to understand and record in clinical practice. According to clinical research, the acceptance of CAD systems with built-in interpretable modules by doctors has increased from 58 to 83%, which significantly promotes the clinical transformation of CAD systems.

This figure illustrates a pipeline for deriving cancer-specific gene signatures from TCGA RNA-seq data. Protein-coding genes with FPKM ≥ 5 in at least 50% of samples were selected, yielding 13,250 genes across 37 cancers. Data were randomly split into training, validation, and test sets to build a deep neural network (DNN) model. SHAP was applied to interpret model outputs, and the top 20 genes per cancer type were identified, generating 370 lists. Genes were retained if their median FPKM ≥ 10, present in at least 5 of 10 lists, and enriched in more than five cancer types. The expression of retained genes was then compared against the other cancers to confirm specificity. This strategy integrates deep learning with statistical filtering to produce robust gene expression signatures for cancer characterization.

## In-depth expansion of clinical application scenarios

### High-risk population screening system

Screening is an important means to reduce the mortality of lung cancer. CAD systems play an irreplaceable role in improving the efficiency and quality of lung cancer screening. In batch LDCT screening, the traditional manual image reading time is 6–8 min per case, while using CAD systems can process 50 cases per hour, increasing efficiency by 8–10 times, and the missed diagnosis rate of micro-nodules with diameter <5 mm after full lung automatic scanning is reduced from 30 to <5%^72^. In the experience of a regional cancer center, the number of lung cancer screenings reached 10,000 cases in the year before the introduction of the CAD system, and 30,000 cases in the year after the introduction, with the early detection rate increased by 27%.

Risk-stratified management of CT-detected pulmonary nodules has moved beyond size-based rules to data-driven, multi-factor triage. In 2024, Nature Medicine reported a triage-driven C-Lung-RADS pipeline that integrated imaging, demographics and longitudinal follow-up from >45,000 exams to refine malignancy risk and streamline downstream work-ups, illustrating how AI can operationalize precision screening in real-world cohorts^73^. In parallel, reader-studies and randomized trials demonstrated tangible clinical effects of assistive AI. In a randomized controlled trial in a health-screening population, AI assistance on chest radiographs increased detection of actionable lung nodules (0.59% vs 0.25%; OR 2.4) without raising false-referral rates—evidence that AI can improve early case-finding without additional harm^74^.

Post-screening risk stratification uses risk stratification models to stratify factors such as nodule size, nodule density, nodule growth rate, age, smoking history, and family history of lung cancer to achieve the goals of annual re-examination for low risk, semi-annual re-examination for medium risk, and clinical biopsy for high risk. The risk stratification model is constructed using multi-factor Logistic regression, with a C-index of 0.89^75^. It can not only avoid overdiagnosis and unnecessary invasive examinations, but also reduce unnecessary biopsies in the screening population by 40% and the incidence of complications such as pneumothorax by 35%.

Longitudinal analysis compares the annual screening images, automatically calculates the nodule volume change rate and density change law, and can detect rapidly growing malignant lesions early. Using 3D registration and volume measurement algorithms, the volume measurement error of the same nodule is <5%, and the VDT calculation accuracy is 90%. Nodules with rapid growth and VDT < 200 days are automatically marked to prompt doctors to give priority to handling, with a sensitivity of 97%, winning opportunities for early intervention. According to multi-center research reports, the CAD-assisted screening system can increase the early lung cancer detection rate by 23% and the 5-year survival rate of the screening population by 15%, fully demonstrating its clinical application value.

### Accurate diagnosis and typing

Clarifying the diagnosis and pathological nature, pathological subtype, and molecular characteristics of lung cancer is the premise of accurate diagnosis and final determination of treatment plans. CAD systems are increasingly used in the identification of pathological subtypes and molecular characteristics of lung cancer. For the identification of common subtypes, a deep learning model can be established based on CT images to achieve an accuracy of 86%^76^; the model performs non-invasive typing by learning the image features of different subtypes, and the consistency with the puncture biopsy results can reach 82%; for rare subtypes, the accuracy of multi-modal data CT + PET can reach 78–82%.

The judgment of the invasion degree by CAD systems directly affects the treatment decision of GGN. The identification of pure ground-glass nodules (pGGN), mGGN, atypical adenomatous hyperplasia, minimally invasive adenocarcinoma, and invasive adenocarcinoma determines whether GGN is observed or surgically resected. GGA system identifies and classifies GGN based on information such as size, proportion of solid components, and boundary shape, with an accuracy of 89%, especially 85% for the diagnosis of minimally invasive and invasive adenocarcinomas. Correspondingly, there is no excessive surgical treatment^77^. Accordingly, the curative effect and postoperative pathological coincidence rate obtained by applying CAD for GGN treatment decisions are 91%.

A cutting-edge application of CAD systems is molecular typing prediction, which correlates radiomics features with driver gene mutation status to guide targeted therapy for patients who cannot undergo biopsy. Based on the correlation between radiomics features and genetic data, a prediction model is established. The AUC value for EGFR mutation prediction is 0.88, 0.85 for ALK fusion, and 0.83 for ROS1 rearrangement. When there is no biopsy sample, it can help doctors select appropriate targeted drugs for patients, allowing some patients to receive effective treatment. For example, a study showed that patients with EGFR positive predicted by CAD received gefitinib treatment, with an objective remission rate of 62%, similar to the efficacy in patients with real mutations.

Recent studies have provided concrete examples of molecular biomarker prediction via imaging, which strengthen diagnostic and therapeutic stratification. For instance, Lu et al. developed a deep learning–radiomics (DLR) model based on CT images to predict PD-L1 expression in NSCLC, with reported AUC = 0.85 (95% CI, 0.82–0.88), and an integrated model (DLR + clinical data) achieving AUC = 0.91^78^. Another work by Zhao et al. constructed a radiomics signature from [^18 F]FDG PET/CT images (n_train = 233, n_val = 101) that noninvasively predicted different PD-L1 expression statuses; in the validation cohort the radiomics model achieved AUC = 0.761 (95% CI 0.664–0.860), superior to the clinical model^79^. Similarly, on the EGFR mutation front, Wu et al. used contrast-enhanced chest CT to build radiomic models predicting EGFR mutation status in NSCLC, reporting AUCs in the range ~0.80–0.90 in multi-institution cohorts^80^. Also, an external validation study by Kohan et al. (2025) on a CT-based radiogenomics model reported strong ability to predict EGFR mutations across diverse patients^67^.

Indeterminate nodule characterization and histologic invasiveness: Multi-center deep learning now supports ternary classification of preinvasive, minimally invasive, and invasive adenocarcinoma directly from chest CT, with adjudication strategies that maintain performance for pGGNs—bridging the gap between imaging appearance and pathology-level decision-making^81^. Radiogenomics (EGFR and beyond): Several studies show robust, externally validated prediction of EGFR mutation status using CT and PET/CT radiomics—often improved further by integrating deep learning feature sets. Representative examples include a 1280-patient CT deep learning–radiomics model in Scientific Reports (noninvasive EGFR prediction) and explainable multi-center PET/CT radiomics that yielded high diagnostic performance and interpretability^82^.

Clinical practice shows that CAD-assisted diagnosis can increase the diagnostic consistency of doctors with different seniority from 65 to 91%, especially narrowing the diagnostic gap between junior doctors and senior doctors, and promoting the standardization of diagnosis.

### Treatment response and prognosis evaluation

**Fig. 6:**
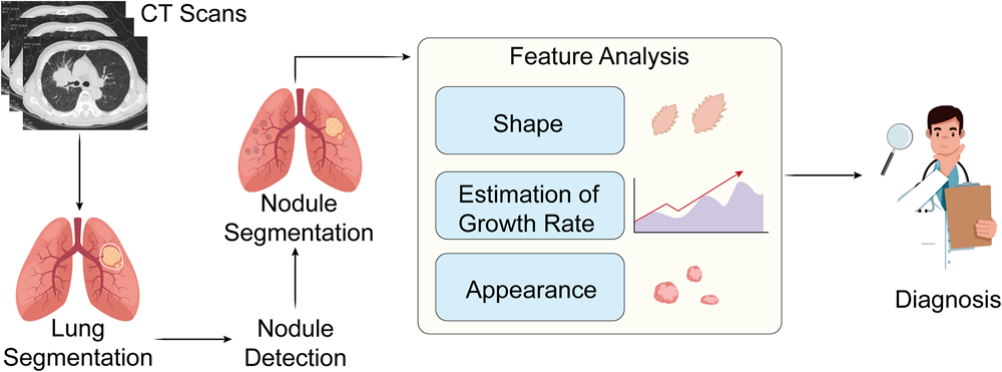
Workflow of lung nodule analysis from CT scans to diagnosis.

CAD systems play an important role in the whole-process management of lung cancer treatment, forming a complete technical support from formulating treatment plans to efficacy evaluation and prognosis prediction. A typical CAD system for lung cancer diagnosis is shown in Fig. 6. In radiotherapy plan optimization, CAD systems use 3D reconstruction of tumor target areas and surrounding organs at risk to accurately estimate the target area volume and target area dose. The target area coverage rate reaches 98%^83^, and the dose to normal tissues is lower than the safe dose. Combined with deep learning automatic target area delineation, the system shortens the target area delineation time from 2 to 3 h manually to 5 min, and the Dice coefficient compared with manual delineation reaches 0.95, increasing the efficiency and consistency of radiotherapy plans.

The efficacy evaluation model compares the quantitative changes in tumor size, density, and metabolic activity before and after treatment to predict treatment response in advance. Especially for immunotherapy, the traditional RECIST standard is often delayed compared with clinical response, while the CAD system predicts the final efficacy in advance based on the “pseudo progression” characteristics of tumors, with an accuracy of up to 84%. In chemotherapy evaluation, the tumor volume reduction rate calculated by the system is correlated with the clinical benefit rate by 0.83, which is more accurate than manual measurement. Response to neoadjuvant immuno-chemotherapy: New CT radiomics work emphasizes that peritumoral as well as intratumoral features predict major pathological response (MPR) pre-treatment, supporting more individualized preoperative strategies for resectable NSCLC. Complementary PET/CT radiomics studies have also reported promising performance for predicting pathologic complete response (pCR) after neoadjuvant therapy, further underscoring the value of multimodal fusion^84^.

CAD combines tumor size, invasion range, lymph node status, age, stage, and treatment plan to establish a nomogram to predict the 1/3/5-year survival rate of patients, with a C-index of 0.87. It can identify high-risk factors for poor prognosis, such as tumor contact area with the chest wall >3 cm² and mediastinal lymph node short diameter >1 cm, and provide individualized treatment strategies. Follow-up confirmed that the adjustment of individualized treatment based on CAD can prolong the median survival of advanced lung cancer patients by 3.5 months and increase the 1-year survival rate by 12%.

This figure shows the pipeline of lung nodule assessment using CT imaging. After lung segmentation, nodules are detected and segmented to define lesion boundaries. Feature analysis is then performed, focusing on shape, growth rate estimation, and appearance. Shape analysis evaluates roundness and spiculation; growth rate is measured by volumetric doubling time across follow-up scans; and appearance considers intensity, texture, and margins. These quantitative features provide key biomarkers for distinguishing benign from malignant nodules. The extracted information supports diagnostic decision-making, enabling risk stratification and guiding treatment planning in lung cancer care.

## Existing challenges and solutions

### Data quality and standardization bottlenecks

The development of lung cancer CAD systems is restricted by data quality and standardization. To solve the problem that the domain shift of ±100HU in the same patient’s image grayscale between different devices due to differences in CT machines from different manufacturers and models reduces the model performance by 10–15%^85^, a device calibration model can be proposed. The GAN-based domain adaptation method learns the style between images from different devices, eliminating the reduction of cross-device diagnostic accuracy caused by different manufacturers to <3%.

Another problem is annotation inconsistency. Different radiologists have inconsistent definitions of the boundaries of fuzzy nodules and understanding of benign and malignant nature, with an annotation coincidence rate of 72%, which affects the training reliability of the model. The expert consensus annotation method can effectively solve this problem. Three physicians with associate senior titles or above annotate independently, and controversial cases are discussed collectively, so that the annotation consistency reaches 90%. In addition, increase annotation quality control indicators, delete or correct annotations that do not meet the quality standards, and improve data quality.

The serious phenomenon of data silos with a multi-center data sharing rate of less than 30% has seriously hindered the collection of large-scale data. Restricted by patient privacy protection and data security regulations, federated learning can break through this bottleneck under the condition of ensuring privacy and security. Medical institutions train models locally, and only share parameter updates between models without sharing raw data. The lung cancer federated learning platform has joined 12 hospitals and completed model training on 10,000 cases of data within 6 months, which is 13% better than the single-center model and fully complies with data privacy regulations. Beyond dataset shift, reproducibility and standardization remain the key barriers to clinical translation of radiomics (feature stability, harmonization, cross-site generalization), as emphasized by recent lung-cancer–focused reviews; this aligns with your discussion of domain shift and further motivates harmonization efforts and multi-center validation^86^.

In addition to highlighting the challenges of data quality, interpretability, and standardization, several practical solutions can be proposed. First, the integration of explainable AI (XAI) approaches can substantially improve model transparency and clinician trust. Methods such as Grad-CAM and SHAP allow visualization of salient regions or feature contributions, thereby making the decision-making process of deep learning and radiomics models more interpretable and auditable. Embedding these explanations into standardized reporting formats (e.g., DICOM SR) also facilitates clinical integration and long-term reproducibility. Second, establishing a multi-center unified radiomics standard database is critical to address heterogeneity across scanners and institutions. Such a repository should include imaging data, segmentation masks, and IBSI-compliant radiomic features, all stored in interoperable formats (e.g., DICOM-SEG, PET-BIDS). Incorporating harmonization strategies such as ComBat, along with rigorous quality assurance protocols and phantom calibration, can further mitigate variability. In parallel, federated learning frameworks may be adopted to enable collaborative model training without direct data sharing, thereby enhancing generalizability while protecting patient privacy.

Together, these strategies provide concrete pathways to overcome current barriers. By combining XAI-driven interpretability with standardized, multi-institutional data infrastructures, lung cancer CAD systems are expected to achieve not only higher accuracy but also improved clinical reliability, reproducibility, and scalability for real-world applications.

### Clinical transformation barriers and regulatory norms

First of all, the clinical transformation of lung cancer CAD systems is full of difficulties. Strict regulatory supervision is the first major obstacle to its clinical transformation. The US FDA conducts risk-level management and control on AI medical devices. AI for lung cancer diagnosis is a high-risk device, requiring submission of more than 1000 cases of verification data, including test results covering various populations and scenarios, and explaining the clinical benefits that the tool can bring. The EU CE certification also controls performance and risk management, requiring complete clinical evidence. These approval processes are long (usually 1–2 years) and costly, limiting the rapid development of new technologies.

Another factor affecting system promotion is the incomplete integration of clinical workflows. At present, the interfaces between the hospital’s PACS system and CAD modules are incompatible, the transmission rate is slow, and the response time of some systems is >10 s, exceeding the clinically acceptable time of <2 s. Solutions include lightweight design of the model, reducing the number of model parameters from 100 M to less than 10 M, which increases the model inference speed by 5 times; and stipulating the interface standards between CAD and PACS, so that CAD can be seamlessly integrated into the PACS system, and doctors can use CAD functions when operating PACS.

Ambiguous responsibility definition is a potential obstacle to clinical application. When the diagnosis result of the CAD system is inconsistent with the pathology^87^, there is no clear standard for responsibility division, leading hospitals and doctors to be cautious about using AI systems.

At present, this problem is developing towards forming a “human-oriented, AI-assisted” decision-making model in the industry. Doctors have the final say on the diagnostic decision, while the AI system only provides reference opinions as an auxiliary means, and reduces the risk of clinical application through the purchase of medical liability insurance and adverse event reporting systems.

At the regulatory level, ISO has issued “ISO/TS13495”, which specifies the requirements for the QMS of AI medical products. China’s National Medical Products Administration (NMPA) has formulated the “Technical Guidelines for the Review of Medical Device Software”, which was released in 2022, stipulating the approval process and technical requirements for AI medical devices, providing regulatory support for the clinical transformation of lung cancer CAD. Worldwide, 12 products of lung cancer CAD systems have been approved by the FDA, and 18 have obtained NMPA certification, and are gradually being applied clinically.

## Future development directions and cutting-edge exploration

### New generation technology trends

Lung cancer CAD systems will develop in the direction of precision, comprehensiveness, and intelligence, with a number of forward-looking technologies emerging. 4D image analysis technology uses respiratory-heartbeat phase information and dynamic modeling to capture the movement trajectory of tumors under different physiological conditions, especially micro-nodules 5 mm and those close to moving organs such as the diaphragm and heart. 4D-CT data uses spatiotemporal convolutional networks to predict tumor movement with an error of <1 mm, which can serve as a more accurate data source for radiotherapy target volume delineation and dose calculation.

Integrating multi-omics analysis is also a hot topic, which will integrate data from radiomics, genomics, proteomics, etc., to construct a “imaging-molecular” joint map. Based on the correlation between CT image tumor enhancement patterns and tumor mutation burden (TMB), it can predict patients’ response to immunotherapy; based on PET tumor metabolic characteristics and PD-L1 expression levels, it can screen beneficial populations. The diagnostic and prognostic value of multi-omics models will be higher than that of single-modal use, and the era of panoramic precision medicine is approaching.

Explainable AI technology will develop from “descriptive explanation” to “causal explanation”. It uses causal reasoning (Do-Calculus) to elaborate the causal relationship between imaging signs and diseases, rather than just correlation. It not only indicates that “spiculation sign” is related to malignant tumors, but also explains the pathological mechanism of “spiculation sign” generation, and that the sign has an independent impact on prognosis and makes an independent contribution to prognosis. Preliminary verification shows that the coincidence rate between the diagnostic reasons of causal AI models and pathological mechanisms reaches 87%, which is significantly higher than 62% of conventional black-box models, which will significantly improve the credibility of the model and its clinical acceptance.

Regional image-sharing alliance (operational blueprint). To translate multi-centric development into routine practice, we propose a regional image-sharing alliance that combines a de-identified DICOM exchange layer with a privacy-preserving learning layer. Concretely, participating hospitals deploy (i) a DICOM router with automatic PHI removal and series-level QA; (ii) a standards-conformant data model using DICOM-SEG for masks and PET-BIDS/PACS metadata to preserve acquisition context; and (iii) a federated-learning node to enable cross-site model training without transferring raw images. Minimum viable data elements include LDCT/diagnostic CT (with reconstruction kernel tags), PET/CT with SUV calibration, RTSTRUCT/RTDOSE (where available), pathology, and a harmonized clinical sheet (age, smoking, stage, treatment). Governance specifies a data-use agreement, site onboarding SOPs, and monthly phantom/QC audits; performance is monitored by predefined KPIs (upload completeness ≥90%, model generalization gap on external sites ≤5% AUC drop, and median curation turnaround <48 h). This design directly addresses data silos while complying with privacy constraints and aligns with our manuscript’s emphasis on multi-center generalization and federated learning.

Interpretability visualization plugin (3D Slicer implementation). To operationalize explainability at the point of care, we recommend a lightweight 3D Slicer extension that (i) ingests a trained model’s saliency maps (e.g., Grad-CAM for CNNs) and feature attributions (e.g., SHAP for radiomics hybrids); (ii) renders synchronized overlays on axial/sagittal/coronal CT as well as PET SUV maps with adjustable opacity and thresholding; and (iii) exports a DICOM Structured Report summarizing the “evidence table” (top features/regions, contribution scores) for archiving and audit. A built-in QA panel should compute region-level fidelity metrics (pointing-game accuracy, deletion/insertion AUC) and log model/version seeds to ensure reproducibility across scanners and sites. Reader-in-the-loop workflows can then quantify changes in decision confidence and reporting time. This plugin path builds directly on the interpretability approaches already discussed in the manuscript and packages them for clinical review and longitudinal traceability.

Radiomics standardization (IBSI-aligned operational checklist). We further propose a pragmatic “LungCAD-RAD-Std” checklist to promote reproducible radiomics across centers: (1) acquisition & preprocessing—record kernel/slice thickness; clip to a lung window and apply Z-score normalization inside a lung mask; (2) resampling & discretization—resample to 1.0–1.5 mm isotropic voxels and use a fixed bin width (e.g., 25 HU), version-locked; (3) segmentation provenance—store masks as DICOM-SEG with rater and tool metadata; (4) feature extraction—use IBSI-aligned PyRadiomics with a YAML configuration under version control; (5) harmonization—apply ComBat (batch = scanner/protocol) with pre/post diagnostics; (6) robustness—report test–retest/scan–rescan ICC and feature perturbation sensitivity, targeting ICC ≥ 0.85 for primary features; and (7) reporting—include calibration and decision-curve analysis alongside AUC/C-index. These steps operationalize our manuscript’s stance on standardization and reproducibility and are consistent with IBSI-aligned tooling already summarized in the paper.

### Expansion of clinical application scenarios

In addition, the application of lung cancer CAD systems will not be limited to lung cancer screening, but will also expand from screening and diagnosis to follow-up. Specifically, such as lung cancer community medical screening: developing a lightweight cloud-based CAD system suitable for community hospitals. The system can rely on mobile devices to obtain CT images transmitted by primary hospitals, conduct real-time AI analysis in the cloud and provide diagnostic reports, so as to improve the lung cancer screening ability of primary hospitals to the level of tertiary hospitals. It should have low-bandwidth adaptability (under 5 G/4 G/3 G environments) and offline analysis capabilities to meet the network conditions in various regions.

Intraoperative navigation is also an important application scenario. Based on intraoperative CT or ultrasound images and preoperative plans, real-time registration technology is used to superimpose and display the tumor location, tumor boundary, and the relationship between the tumor and surrounding blood vessels and nerves in the surgeon’s surgical field of view, so as to improve the accuracy of tumor resection. The system requires sub-millimeter registration accuracy and <1 s delay to ensure surgical safety. Experimental data show that AI-based intraoperative navigation can reduce the positive rate of surgical margins in early lung cancer to <5%, which is much better than 15% in traditional surgery.

Regular review results can be uploaded through a mobile phone APP on the mobile terminal, and the AI system can intelligently compare the size and density of nodules regularly through historical follow-up images. Through automatic follow-up suggestion reminders, recording patients’ symptom information and importing living habits into the system, and providing personalized health guidance according to different patients’ conditions, the patients’ follow-up compliance is improved, so as to achieve early detection of diseases.

By 2030, lung cancer CAD systems will cover the entire process of “screening-diagnosis-treatment-follow-up”, and be deeply integrated with electronic medical record systems and laboratory information systems to form an intelligent closed-loop of lung cancer diagnosis and treatment. The early diagnosis rate of lung cancer will reach more than 60%, and the 5-year survival rate will increase to more than 50%, which will greatly promote the reduction of lung cancer mortality.

## Conclusion

After more than 30 years of unremitting efforts, lung cancer computer-aided diagnosis systems have developed from initial auxiliary tools to intelligent technical systems covering lung cancer diagnosis, treatment and prognosis. The core technologies of lung cancer CAD have evolved from traditional algorithms and machine learning to deep learning, from manual features to automatic features, from single-modal to multi-modal, and from diagnosis to prognosis. Lung cancer CAD has made lung cancer screening, diagnosis accuracy and treatment personalization more efficient in clinical applications, and has also played a positive role in improving the prognosis of lung cancer patients.

However, it still faces problems such as data quality and standards, model interpretability, and clinical transformation barriers, which need to be solved by joint efforts of technology, systems, industry, university and research institutions. It is believed that with the further maturity of technologies such as multi-omics integration, 4D image analysis, and explainable AI, lung cancer CAD systems will better play the role of precision medical assistance in the future, truly bringing lung cancer diagnosis and treatment into a new era of intelligence and individualization. On the one hand, scientific research teams and clinical medical staff should work together, guided by real clinical needs, pay close attention to technological innovation and transformation applications, and finally make lung cancer CAD systems a driving force for improving the level of lung cancer diagnosis and treatment, benefiting patients.

## Data Availability

No datasets were generated or analysed during the current study.
